# Electrical cardioversion

**DOI:** 10.4103/0256-4947.51775

**Published:** 2009

**Authors:** Murat Sucu, Vedat Davutoglu, Orhan Ozer

**Affiliations:** From the Department of Cardiology, Gaziantep University School of Medicine, Gaziantep, Turkey

## Abstract

External electrical cardioversion was first performed in the 1950s. Urgent or elective cardioversions have specific advantages, such as termination of atrial and ventricular tachycardia and recovery of sinus rhythm. Electrical cardioversion is life-saving when applied in urgent circumstances. The succcess rate is increased by accurate tachycardia diagnosis, careful patient selection, adequate electrode (paddles) application, determination of the optimal energy and anesthesia levels, prevention of embolic events and arrythmia recurrence and airway conservation while minimizing possible complications. Potential complications include ventricular fibrillation due to general anesthesia or lack of synchronization between the direct current (DC) shock and the QRS complex, thromboembolus due to insufficient anticoagulant therapy, non-sustained VT, atrial arrhythmia, heart block, bradycardia, transient left bundle branch block, myocardial necrosis, myocardial dysfunction, transient hypotension, pulmonary edema and skin burn. Electrical cardioversion performed in patients with a pacemaker or an incompatible cardioverter defibrillator may lead to dysfunction, namely acute or chronic changes in the pacing or sensitivity threshold. Although this procedure appears fairly simple, serious consequences might occur if inappropriately performed.

External electrical cardioversion (EEC) was first performed in the 1950s.^1^ This early experience demonstrated that electrical energy externally delivered to the thorax could stimulate the heart. In 1955, Zoll et al successfully terminated ventricular fibrillation in a patient with externally applied defibrillation shocks. Kouwenhoven and colleagues discovered that a battery-powered direct current operated device could be fully portable.^2^ Electrical countershocks can be delivered with either alternating current (AC) or direct current (DC) energy. Alternating currents are sinusoidal energy waveforms that switch between positive and negative polarity. The energy pulse lasts for approximately 200 ms once discharged. Alternating current defibrillation can cause significant myocardial damage due to the greater energy flux and duration.^3^ Urgent or elective DC cardioversions have specific advantages, such as termination of atrial and ventricular tachycardia and recovery of sinus rhythm.

## Mechanism of external cardioversion and defibrillation

The current external electrical cardioversion technique relies on the application of a selected amount of energy, which is generally between 50-360 J, via two electrodes (paddles). The mechanism of defibrillation is not known exactly. Zipes et al have suggested that failure to maintain the reentrant tachycardia by the remaining myocardial tissue after depolarization of a critical mass is the main factor in the mechanism of electrical defibrillation.^4^ Another research group has suggested that the shock waves of defibrillation prolong refractoriness in a sufficient mass of myocardial tissue and, consequently, terminate ventricular fibrillation (VF).^3^ Whether the mechanisms suggested for the termination of VF are similar to those of atrial fibrillation (AF) remains unknown.

A lower limit of vulnerability also exists for the ventricular myocardium. This is the minimum voltage required by an electrical stimulus to induce fibrillation during the vulnerable period. It was also observed that the strengths of these shocks at “the upper limit of vulnerability” were approximately equivalent to the shocks at defibrillation threshold. The upper limit of vulnerability hypothesis for defibrillation states that to defibrillate, a shock not only must halt the activation fronts of fibrillation, but it also must not reinitiate fibrillation by the same mechanism that a shock of the same strength during the vulnerable period of sinus or paced rhythm initiates fibrillation.^5^

## Indications

### Atrial fibrilation and atrial flutter

Currently, electrical cardioversion is mostly performed to convert AF and atrial flutter into sinus rhythm. AF, encountered in intensive care units after cardiac surgery, is not suitable for electrical cardioversion since it is paroxysmal. In the treatment of recurrent AF attacks, prevention of trigger mechanisms, application of specific antiarrhythmic therapies and improvement of electrolyte imbalance and hypoxia avert inappropriate cardioversion applications by blocking such episodes. AF treatment, on the other hand, includes anticoagulant therapy to minimize the risk of embolus and stroke during elective cardioversion. If AF persists for longer than 48 hours, anticoagulant therapy for 3 weeks is recommended with the international normalized ratio (INR) maintained >2.0. In case atrial activity is not achieved, therapy should be extended for 4 weeks, even after restoring sinus rhythm by electrical effective cardioversion.^6^ A shorter-lasting AF (<48 hours) can alternatively be treated by heparinization and monitored by transesophageal echocardiography (TEE) prior to the cardioversion.^7^ Any thrombotic formations within the left atrial appendix can be detected by TEE. The risk/benefit ratio should be assessed in all patients pribb or to cardioversion. Not observing a thrombus in TEE does not exclude the risk of embolus.

### Venctricular and supraventricular tachycardia

Emergency electrical cardioversion is performed in unstable ventricular tachycardia that causes hemodynamic deterioration. If a patient with ventricular tachycardia is hemodynamically stable, sinus rhythm should be achieved by intravenous antiarrhythmics. Anticoagulation therapy is usually not necessary for cardioversion of ventricular tachycardia. Emergency electrical cardioversion is performed in unstable ventricular tachycardia that causes hemodynamic deterioration. The aim of ventricular tachycardia treatment is the prompt termination of this impairment. Electrical cardioversion should be the next step in case of unsuccessful antiarrhythmic treatment and vagotonic manoeuvres such as carotid sinus massage for sustained supraventricular tachycardia. The success rate with cardioversion of ventricular tachycardia is generally around 95%.^8^

### Other rhythm disorders

Electrical shock treatment is ineffective in the termination of automaticity related tachycardies. Multifocal atrial tachycardias of this kind are generally confused with AF, which may lead to the inappropriate application of direct current (DC) shocks. Since electrical cardioversion may cause resistant VF in patients with digitalis toxicity, it is contraindicated in the presence of this condition.^8^ Atrial flutter and ventricular tachycardia are tachycardias maintained by reentry circuits that may be terminated by local depolarization in the path of their circulating wavefronts.^8^

## Factors affecting EEC success

Energy is a combination of voltage and current. The overall success of the electrical cardioversion is related to the size of electrodes (paddles), application site, transthoracic impedance, and the applied current. For a given amount of energy, the amount of current reaching the myocardium depends in part on the electrical impedance between the two defibrillator paddles. The distance between electrodes, electrode-skin interface and pressure (air is a poor conductor of electrical current), previous shocks (reduced impedance), time span between shocks (3-minute wait may cause resistance reduction), and physical characteristics (tall stature or increased BMI) decrease the rate of success.^9^ The longterm success for AF is associated with the arrhythmia duration (>1 year) and the diameter of the enlarged left atrium (>5 cm).^8^ Recurrence is more frequent in patients with untreated hyperthyroidism, mitral stenosis or congestive heart failure. Successful cardioversion is dependent on certain factors, of which time might be the most important one.^10^ Since myocardial adenosine increases the defibrillation threshold, which is dependent on VF duration, its enhanced release may have a deleterious effect on defibrillation.^11^ Another suggestion is that local changes in potassium concentration may increase defibrillation threshold.^12^ If fibrillation time is prolonged in patients with AF, both postrepolarization and conduction delay occur due to global ischemia.^13^ Energy is a combination of voltage and current.

### Electrodes position

Defibrillator paddles can be used in different configurations, which affect the success rate of defibrillation. According to the International Liaison Committee on Resuscitation (ILCOR) guidelines, the sternal paddle should be placed ‘just to the right of the upper sternal border below the clavicle and the apical paddle to the left of the nipple with the centre of the electrode in the midaxillary line.^14^ Anteroposterior (parasternal and left infrascapular) positioning should be preferred for unsuccessful applications. It holds lower transthoracic impedance. By placing the paddles onto the chest wall, more current is delivered to the atria.

### Thoracic impedance

Thoracic impedance is another important factor in treatment by electrical cardioversion. The paddle area is the main determinant of transthoracic impedance. Current is inversely related to impedance. The optimal paddle size varies between 8 cm to 12 cm. Air between the chest wall and the paddles prevents the effective conduction of the shock wave by increasing transthoracic impedance. Any conductive material between paddles, such as gel or similar materials, increases conductance and decreases transthoracic impedance between the chest wall and the metal electrodes. Either special adhesive pads or special gels are used for this purpose. Contact between paddle area and chest wall must be provided. Gel between paddles should not touch in order to prevent current disorientation.^15^ Such simple precautions cause a decrease in transthoracic impedance and increase the success of shock therapy. The resistance between electrode and the skin enhances the efficacy of electrical cardioversion therapy by providing appropriate pressure on impedance paddles. Transthoracic impedance decreases if the pressure applied on the defibrillator paddles is increased, if appropriate contact between paddles and chest wall is provided, and expiration period is less resistant compared to the inspiration. The shock wave is transmitted during the expirium period.^16^

### Electrode size

In pediatric patients ≤10 kg, pediatric paddles should be used for electrical cardioversion, with an applied pressure of 2.9 kgf. If the child is >10 kg, adult-type paddles should be used, with an applied pressure of 5.1 kgf.^17^ There are reports suggesting that chest shaving adds to the success of electrical cardioversion.^13^ Sado et al stated that chest hair increased transthoracic impedance, which could be reduced up to 35% by shaving. It is recommended to shave the chest hair of such patients prior to defibrillation.^18^

### Medications and sedations

In a pilot study, Sutton et al demonstrated that atropine administration increased the success rate of direct current cardioversion for atrial fibrillation.^19^ Electrical cardioversion together with antiarrhythmics (such as amiodarone) may help in the maintenance of sinus rhythm. It has been demonstrated that verapamil treatment prior to defibrillation may prevent recurrence of early AF by affecting atrial remodeling.^20^ A recent study showed that short-term verapamil treatment associated with propafenone or amiodarone seems to be useless for the prevention of recurrent AF after low energy intracardiac cardioversion.^21^ Quinidine and propafenone seem to be effective in preventing immediate reinitiation of AF. Ibutilide may prevent shock failure, although neither ibutilide nor dofetilide seems to be effective in preventing immediate reinitiation of AF.^22^ For refractory ventricular fibrillation or pulseless ventricular tachycardia, intravenous amiodarone 300 mg can be given initially, followed by a second dose of 150 mg. As an alternative, intravenous lidocaine 1-1.5 mg may be given, followed by 0.50-0.75 mg doses as needed up to a maximum of 3 mg.^23^

Patients may feel pain even at low energy levels (1 J). Electrical cardioversion with high levels of energy should never be performed in conscious patients, since it may cause lifelong emotional disorders and psychological trauma.^24^ Administration of benzodiazepines, either alone or together with opiates, is not recommended in patients who will undergo electrical cardioversion. Propofol, an intravenous agent, seems to be an ideal drug for anesthesia since it acts rapidly, causes less laryngospasm, and loses its effect as soon as the treatment is ceased.^25^ For elective EEC, deep sedation or anesthesia is required in stable patients. Mild sedation is sufficient for less painful procedures.^26^

## Energy selection

### Biphasic shock and monophasic shock

Transthoracic monophasic defibrillators have been employed for the management of ventricular arrhythmias. European Resuscitation Council guidelines recommend a 200 J, 360 J sequence with subsequent shocks at 360 J if the arrhythmia is uncorrected.^14^ Recent studies of ventricular fibrillation have confirmed the superiority of various biphasic waveforms over monophasic pulses of quivalent or similar duration. Biphasic waveforms offer equivalent or superior efficacy at lower energy and peak voltage than their monophasic counterparts.^27^ Biphasic waveform shocks with a fixed energy of 150 J were as effective as conventional sequential monophasic waveform with progressive energy levels of 200, 300 and 360 J for successful defibrillation. However, the low-energy biphasic waveform shocks significantly decreased the severity of postresuscitation myocardial dysfunction.^28^

An initial energy level for AF of 100-200J is recommended. The success rate of electrical cardioversion is approximately 50%. In a recent study, an initial energy level of 360 J was suggested for persistent AF lasting more than 48 hours.^29^ In a study comparing 50 J with 100 J for the conversion of atrial flutter to sinus rhythm, 100 J was considered the best initial energy level.^30^ Energy set between 100-200 J is recommended for monomorphic ventricular tachycardia, although lower energy levels may be effective as well.^31^ On the other hand, the initial defibrillation threshold is suggested to be 200 J in polymorphic ventricular tachycardia or VF. The energy level should be 1-2 J/kg in children with ventricular tachycardia without a pulse or VF.^14^ Low energy levels produce lesser myocardial damage and post-resuscitation myocardial dysfunction.^28^ Synchronization with R wave is mandatory to prevent resistant VF that could be induced by shock wave and T wave overlapping, called a vulnerable period, if a QRS complex appeared during electrical cardioversion. Careful electrocardiographic (ECG) evaluation is required to avoid inappropriate shock therapies in patients with sinus or automatic tachycardia. Shock therapy should not be performed if only one derivation is being monitored in hemodynamically stable patients. Recurrent inappropriate and ineffective shock therapies can have dangerous consequences. Pharmacological prevention of atrial flutter recurrences is quite difficult. The typical recurrent atrial flutter should be treated by catheter ablation. The presence of a structural cardiac anomaly together with continuous ventricular tachycardia or VF holds a risk for sudden death. An implantable cardioverter defibrillator (ICD) should be considered if there is no identifiable cause. Internal cardiac cardioversion is safe and effective in patients with resistant AF.^32^ A study comparing the efficacy of external and internal cardioversion in AF showed that internal cardioversion was more effective in restoring sinus rhythm.^33^ Loss of QRS voltage after EEC or defibrillation was suggested to be of electrical origin or consequent to an edematous area on the chest wall due to trauma. Another proposed reason is myocardial stunning.^34^

## Management of patients with failed EEC or resistant patients

The risks and benefits of recurrent electrical shock therapy must be taken into account. Unnecessary recurrent DC shock treatment should be avoided. The upper shock limit applied by several defibrillators currently used is 360 J, and the waveform is monophasic. As technology progresses, biphasic shock waves are employed. ICDs are the best example of such application. The transvenous activity, the accessible implantation, and the smaller dimensions have contributed to the wide therapeutic adoption of ICD devices. Lown mentioned that higher energy levels were required to terminate AF in congestive heart failure. Restoration of cardiac compensation and achievement of a dry weight before cardioversion increased the success rate. In cases of polycytemia, it may be difficult or impossible to revert until adequate phlebotomy lowers the hematocrit to less than 50. Patients with severe mitral valve disease having giant scarred atria who have had valve repair or replacement are recalcitrant to cardioversion and do not persist in sinus rhythm.^31^ The rate of sinus rhythm re-establishment via internal cardioversion in AF, which is resistant to 360 J, varies between 70% and 80%.^35^ The use of biphasic waves through multipolar catheters, which are placed within the right atrium and coronary sinus, enhances the success rate of cardioversion by markedly reducing the required energy. Despite these low energy levels (2-3 J), sedation and anesthesia are necessary because of the painful procedure.

In new devices, the electrodes are placed within the right atrium and the left pulmonary artery for internal cardioversion. Superior homogeneous electrical dispersion and cardioversion effectiveness have been reported with such electrode placement.^9^ In a multicenter study by Bardy et al, it was reported that automatic external defibrillators, which use biphasic wave shocks, required lower energy than those devices using monophasic waves.^36^ A biphasic shock waveform is known to reduce the ventricular defibrilation testing.^37^ Since synchronization with the R wave is not feasible for these devices, they are not applied in regular tachycardia with R waves.

## Complications and contradictions of cardioversion

Complications are minimal. Potential complications include VF due to general anesthesia or lack of synchronization between the DC shock and the QRS complex, thromboembolus due to insufficient anticoagulant therapy, non-sustained VT, atrial arrhythmia, heart block, bradycardia, transient left bundle branch block, myocardial necrosis, myocardial dysfunction, transient hypotension, pulmonary edema and skin burn. Pain at the application site is associated with the number of applications.^38^ EEC is contraindicated in case of the presence of left atrial thrombus and insufficient anesthesia in stable patients.

## Special conditions

### Arrhythmias in intensive care patients

Atrial and ventricular tachycardias are frequent in patients being treated at intensive care units due to the presence of multiple triggers. Hypoxia, endogenous or exogenous catecholamines, congestive heart failure, fever and pulmonary embolus are particular causes of tachycardia. Patients unable to receive the drugs orally or with poor absorption are more prone to side effects, such as hypotension, which may especially occur with intravenous drugs like amiodarone. Rapid and through-out examination prior to the electrical cardioversion is important. Improvement of trigger and underlying etiologic factors enhances the success rate of cardioversion.^8^ It should be kept in mind that many consecutive shocks and anesthesia in recurrent doses may worsen hemodynamic status in those patients.

Internal cardiac cardioversion is safe and effective in patients with resistant AF.^34^ A study comparing the efficacy of external and internal cardioversion in AF showed that internal cardioversion was more effective in restoring sinus rhythm.^35^

### Cardioversion in Pregnancy

Some investigators have reported that electrical cardioversion is safe during pregnancy.^39^ Cardioversion between 50 J and 300 J applied at various pregnancy phases revealed negligible effects on the fetus, which means harmful electrical current may not reach the fetus.^40^,^41^ Almost 100 years ago, in his animal experiments, Garrey emphasized that a critical myocardial mass would be required for VF. For this reason, it can be assumed that cardioversion may not affect the fetus.^42^

### EEC in patients with pacemakers

Electrical cardioversion performed in patients with a pacemaker or ICD may lead to dysfunction, namely acute or chronic changes in the pacing or sensitivity threshold.^43^ If a patient with a pacemaker should undergo cardioversion, the function and leads of the device should be checked, along with high voltage programming. Pacemaker mode should be switched to VOO or AOO if suitable. During cardioversion, the defibrillator paddles should be placed at a minimum of 15 cm apart from the pacemaker. They should be positioned perpendicularly to the anterolateral, anteroposterior or endocardial leads. A 5-minute interval between two shock wave applications is required. The pacemaker battery and lead functions must also be checked after cardioversion. A high threshold should be maintained for at least 4-6 weeks. In case of pacemaker and lead dysfunction, lead replacement is the advised procedure.^43^ During cardioversion, patients with a pacemaker or ICD may be exposed to burns in the myocardial tissue where the pacemaker lead is attached if electrical current is delivered through it.

## Conclusions

Cardioversion/defibrillation equipment is of the utmost importance to clinicians. Electrical cardioversion is a life-saving when applied in emergency circumstances. In elective cardioversion, accurate tachycardia diagnosis, careful patient selection, adequate electrode (paddles) application, determination of the optimal energy and anesthesia levels, prevention of embolic events and arrythmia recurrence, and airway conservation increase the success rate while minimizing possible complications. Although this procedure appears fairly simple, serious consequences might occur if inappropriately performed.
